# Demographic and clinical characteristics of children with autosomal dominant polycystic kidney disease: a single center experience

**DOI:** 10.3906/sag-2009-79

**Published:** 2021-04-30

**Authors:** Belde KASAP DEMİR, Fatma MUTLUBAŞ, Eren SOYALTIN, Caner ALPARSLAN, Merve ARYA, Demet ALAYGUT, Seçil ARSLANSOYU ÇAMLAR*, Afig BERDELİ, Önder YAVAŞCAN

**Affiliations:** 1 Department of Pediatrics, Division of Nephrology and Rheumatology, İzmir Katip Çelebi University, İzmir Turkey; 2 Department of Pediatrics Division of Nephrology, Tepecik Training and Research Hospital, Health Sciences University, İzmir Turkey; 3 Department of Molecular Medicine, Ege University, İzmir Turkey

**Keywords:** Autosomal dominant polycystic kidney disease, children

## Abstract

**Background/aim:**

In children with autosomal dominant polycystic kidney disease (ADPKD), clinical manifestations range from severe neonatal presentation to renal cysts found by chance. We aimed to evaluate demographic, clinical, laboratory findings, and genetic analysis of children with ADPKD.

**Materials and methods:**

We evaluated children diagnosed with ADPKD between January 2006 and January 2019. The diagnosis was established by family history, ultrasound findings, and/or genetic analysis. The demographic, clinical, and laboratory findings were evaluated retrospectively. Patients <10 years and ≥10 years at the time of diagnosis were divided into 2 groups and parameters were compared between the groups.

**Results:**

There were 41 children (M/F: 18/23) diagnosed with ADPKD. The mean age at diagnosis was 7.2 ± 5.1 (0.6–16.9) years and the follow-up duration was 59.34 ± 40.56 (8–198) months. Five patients (12%) were diagnosed as very early onset ADPKD. All patients had a positive family history. Genetic analysis was performed in 29 patients ( *PKD1* mutations in 21, *PKD2* mutations in 1, no mutation in 3). Cysts were bilateral in 35 (85%) of the patients. Only one patient had hepatic cysts. No valvular defect was defined in 12 patients detected. Only 1 patient had hypertension. None of them had chronic kidney disease. No difference could be demonstrated in sex, laterality of the cysts, maximum cyst diameter, cyst or kidney enlargement, follow-up duration, or GFR at last visit between Groups 1 and 2.

**Conclusion:**

The majority of children with ADPKD had preserved renal functions and slight cyst enlargement during their follow-up. However, they may have different renal problems deserving closed follow-up.

## 1. Introduction 

Autosomal dominant polycystic kidney disease (ADPKD, previously termed adult polycystic kidney disease) is the most common hereditary renal disorder [1]. It is characterized by cystic dilations generating from any part of the nephron with enlarged kidneys and occurs in 1:1000 live births [1,2]. The disease is genetically heterogeneous and the genes involved are *PKD1* , located on chromosome 16p13.3, and *PKD2* , located on 4q21, encoding polycystin-1 and -2, respectively [1,2]. 

Most patients with ADPKD present as adults; however, the timing of cyst generation may be discrete leading to the antenatal period. Cysts enlarge by age. Cyst burden may be significant in childhood and renal failure typically develops in the sixth decade [1]. Why some cases of ADPKD present in childhood is unclear, because there is no evidence for genetic heterogeneity [2]. Screening and monitoring is controversial in the childhood [2].

Polyuria/polydipsia is usually the first biological renal manifestation, which is associated with decreased concentrating ability [1,2]. Other renal manifestations include flank pain, hematuria, proteinuria, hypertension, nephrolithiasis, urinary tract infections, infection of the cysts, or hemorrhagia into the cysts [1–3]. It is a systemic disorder and extrarenal manifestations including hepatic, pancreatic or seminal vesicle cysts, cerebral aneurysms, aortic aneurysms and dissection, coronary artery aneurysms, colonic diverticula, abdominal wall and inguinal hernia and cardiac valve disease may occur [1,2]. Although they are well-defined in adults, diagnostic criteria and management consensus are uncertain in children. 

In this study, we evaluated demographic, clinical, laboratory findings, and genetic analyses of our patients with ADPKD. We aimed to report familial burden, genetic contribution, cyst characteristics, renal and extrarenal manifestations, treatment options and follow-up modalities in a series of children being followed up in our clinic for a relatively long period and demonstrated whether these parameters differed between cases diagnosed in the first decade or thereafter. 

## 2. Materials and methods

We evaluated children diagnosed with ADPKD between January 2006 and January 2019. The diagnosis was established by family history, ultrasound findings, and/or genetic analysis. The demographic, clinical, and laboratory findings including age at diagnosis, sex, follow-up duration, parental inheritance pattern, the number of people diagnosed with ADPKD in the family, renal manifestations including laterality of cysts, number and diameter of the largest cysts at first and last visit in each kidney on ultrasound, extrarenal manifestations, evidence for hypertension (HT), kidney stone, urinary tract infections (UTIs), hematuria, proteinuria or pyuria, GFR at the last visit and genetic mutations (if available) were evaluated retrospectively. Children diagnosed in utero or within the first 18 months of life were grouped as very early onset (VEO) ADPKD [2]. Genetic analysis has been performed by Sanger sequencing until a few years ago. Only PKD1 mutation could have been defined by this method. Then, next-generation sequencing went into use for screening both *PKD1* , *PKD2,* and *PKHD1* mutations.

Cyst enlargement per month (CEM) was estimated by the formula: Δ cyst diameter (diameter of the largest cyst at last visit – diameter of the largest cyst at first visit/follow-up duration). Kidney enlargement per month (KEM) has been determined for both right and left kidneys by the following equation: Δ kidney length (kidney length at last visit – kidney length at first visit)/follow-up duration. The highest KEM was recorded. Hypertension was defined as blood pressure ≥95 percentile for height and sex. Pyuria was defined as ≥5 white blood cells/mm3 and microscopic hematuria was defined as ≥5 red blood cells/mm3. Proteinuria was defined as a protein/creatinine ratio >0.21 in the spot urine sample or ≥4/mg/m2/h in 24-h collected urine. The estimated glomerular filtration rate (eGFR) was calculated due to the Schwartz formula as follows: eGFR = k x height in cm/serum creatinine. Since the serum creatinine is determined by Jaffe’s reaction in our laboratory, we used 0.55 for girls and boys younger than 13 years and 0.7 for boys ≥13 years for constant k, which is directly proportional to the muscle component. Glomerular hyperfiltration (GHF) was defined as GFR ≥140 mL/min/1.73 m2 [4].

Patients were divided into 2 groups due to their age at diagnosis arbitrarily. Patients <10 years at the time of diagnosis were grouped as Group 1 to present patients diagnosed in the first decade. Patients, those ≥10 years were diagnosed as Group 2. The parameters described above were compared between the groups. 

Statistical analyses were performed using IBM SPSS 22.0 (IBM Corp., Armonk,NY, USA). Kolmogorov–Smirnov test was used to evaluate the normal distribution of continuous variables between the groups. The continuous variables were presented as either mean ± standard deviation or median (25p–75p) according to data distribution and categorical variables were presented as percentage. The parameters with normal distribution were compared by Student’s t-test, whereas those without normal distribution were evaluated by Mann–Whitney U test. The categorical variables between the groups were compared using the chi-square test. Depending on the distribution type of the variables, Pearson or Spearman correlation analysis was performed. A P value of <0.05 was considered significant in all statistical evaluations.

## 3. Results

There were 41 children diagnosed with ADPKD in the mentioned period. Of those patients, 18 (44%) were male and 23 (56%) were female. The mean age at diagnosis was 7.2 ± 5.1 (0–16.9) years. Five patients (12%) were diagnosed as VEO ADPKD. The mean follow-up duration was 59.34 ± 40.56 (8–198) months. A family history of ADPKD was known at admission in all patients. Inheritance was maternal in 20 (49%) and paternal in 21 (51%) of them. Twenty (49%) of the patients were siblings belonging to 9 families. The mean number of patients diagnosed with ADPKD in the family were 4.33 ± 1.81 (1–7). 

Genetic analysis could have been performed in 29 (70%) of the patients (Table 1). In 4 of them, *PKD1* mutation was found by Sanger sequencing. In the rest of the group detected by next-generation sequencing, no mutation could be detected in 3 patients, while 1 had *PKD2* mutation and 21 had *PKD1* mutation. Of the 5 VEO patients, 2 had in utero cysts and early diagnosis as their parents had the disease and requested screening for ADPKD. Of the latter two, one had a stop codon mutation, while the other had a missense mutation (Table 2).

**Table 1:** Mutations in children with ADPKD.

Patients	Mutations
P1	p.L727P (c.2180T>C)
P2	Ala3281Thr (c.9841G>A)
P3-5 (F1)	p.Glu2771Lys (c.8311G>A)
P6-7 (F2)	p.Asp3451Asn(c.10351G>A)
P8-10 (F3)	p.Phe2979del (c.8935-8937delTTC)
P11	No mutation
P12	p.Val2334fs (c.7000_7001insGCTGGCG)
P13	p.Gly3144Ala(c.9431G>C)
P14	p.Arg325X (c.973C>T)*
P15-16 (F4)	p.Thr579Met (c.1736C>T) ( Heterozygous)
P17-18 (F5)	p.Pro2258Arg (c.6773C>G)(Heterozygous)
P19-20 (F6)	p.Leu1486fs (c.4454_4455insTA)
P21	3793GlyfsX3826
P22	Thr3510Met
P23-24 (F7)	3791SerfsX3814
P25	p.Glu3518X (c.10552 G>T)
P26	p.Glu2771Lys (c.8311 G>A)
P27	p.Arg4154Cys (c.12460 C>T)
P28	No mutation

P: patient, F: family, *: mutation in PKD2 gene.

**Table 2:** Characterization of patients with very early onset

	Sex	Time the first cystdetermined (month)	Family history	Mutation
P6	F	17	Mother, siblings	p.Asp3451Asn(c.10351G>A)*
P11	M	AN	Father	No mutation in PKD1, PKD2 or PKDH1
P24	F	AN	Mother	p.Glu3518X (c.10552 G>T) **
P27	M	AN	Mother	p.Arg4154Cys (c.12460 C>T)*
P29	M	6,5	Mother	ND

AN: antenatal, ND: not determined, *: missense mutation, **: nonsense stop codon mutation.

Renal cysts were bilateral in 35 (85%) of the patients. The remaining three had cysts on the left and three had cysts on the right kidney at the first visit and three of them had bilateral cysts at last visit. The mean diameter of the largest cyst at the last follow-up was 14.49 ± 9.5 (2–60) mm. The calculated CEM was 0.19 ± 0.2 (–0.13–0.77) and KEM was 0.31 ± 0.13 (–0.15–0.54). Only one patient had hepatic cysts, and none of them had splenic or pancreatic cysts. Cranial magnetic resonance angiography was performed in 5 patients and none had an aneurysm. Echocardiography was performed in 12 of the patients that were normal in 11 and demonstrated PFO in one of them.

Recurrent UTIs were detected in 6 (14%), HT was observed in 1 (2%), urolithiasis was defined in 3 (7%), proteinuria in 6 (15%), pyuria in 4 (10%), and hematuria in 5 (12%) of the patients. None had chronic kidney disease in the follow-up and mean GFR at the time of the last visit was 127.3 ± 21.8 (89.8–172) mL/min/1.73m2. No difference could be demonstrated in sex, laterality of the cysts, maximum cyst diameter, CEM, KEM, follow-up duration, or GFR at last visit between Groups 1 and 2 (Table 3). Besides, there was no significant correlation between the GFR values at the last visit and the maximum cyst diameter (r = –0.236, P = 0.187), CEM (r = –0.225, P = 0.231), KEM (r = 0.193, P = 0.377) or follow-up duration (r = –0.018, P = 0.923). 

**Table 3:** Demographic, radiological and clinical differences due to age at admission.

	< 10y at admission (n = 30)	≥ 10y at admission (n = 11)	P
Sex (female)	17 (57%)	6 (55%)	0.961
Laterality (bilateral)	25 (83%)	10 (91%)	1.000
Max. cyst diameter at first admission (mm)	12.76 ± 5.49 (2–20)	18.09 ± 14.51 (10–60)	0.174
Max. cyst enlargement rate (mm/month)	0.11 (0.35–0.19)	0.33 (0.16–0.47)	0.082
Max. kidney enlargement rate (mm/month)	0.31 (0.24–0.38)	0.39 (0.25–0.44)	0.522
Follow-up duration (months)	61 (35–82.5)	24 (18.5–64.5)	0.176
GFR at last visit (mL/min/1.73m2)	125.61 ± 23.32 (89.83–171.87)	131.22 ± 18.04 (100–172)	0.808
GHF at last visit	9 (31%)	2 (18%)	0.457

GFR: glomerular filtration rate, GHF: glomerular hyperfiltration.

## 4. Discussion

Autosomal dominant polycystic kidney disease is the most common monogenic renal disorder causing renal failure all over the world [1,2,5]. Although end-stage kidney disease is expected in the sixth decade and it is controversial to diagnose at-risk children, patients diagnosed with ADPKD in childhood are becoming more common. In this study, we reviewed our 41 patients with ADPKD diagnosed in childhood. Five of them were diagnosed in the first 18 months of their lives, so called “VEO” ADPKD with two being diagnosed in utero. 

 In ADPKD, clinical manifestations range from severe neonatal presentation to renal cysts found by chance in the childhood period [2]. Why some ADPKD cases present in childhood is unknown and the type of mutation was thought to be the underlying factor. It is well-known that patients with a truncating mutation are more severely affected [2]. Children diagnosed as VEO ADPKD also represent a high-risk group. They are known to have more HT and progression to chronic kidney disease (CKD) at a median age of 16 years [2]. Five of our patients (12%) were diagnosed with VEO ADPKD. Mothers of all five children were also diagnosed with ADPKD and one also had a sister with the disease. Two of them had an antenatal diagnosis due to cysts determined in utero. Of the latter two cases, five and four other relatives except their mothers were diagnosed with ADPKD, respectively. Both had bilateral multiple cysts with the largest diameter of >10 mm in the antenatal and early postnatal periods. The first case had a nonsense mutation resulting in a stop codon, while the other case had a missense mutation. 

The genes responsible for ADPKD are *PKD1* or *PKD2,* encoding polycystin-1 (PC1) and -2 (PC2), and accounting for 85 and 15% of the patients, respectively [1,2]. Recently, a third gene, namely *GANAB* (glucosidase, alpha, neutral AB form) *,* has been identified to cause ADPKD [2]. Most cases are familial, however, family history is lacking in 10%–25% of patients including de novo disease in up to 10%–15% [6]. In our series, all the cases had a maternal or paternal history of ADPKD and none had a de novo condition at all. Twenty-five patients had *PKD1* and only 1 patient had *PKD2* mutation. Unfortunately, *PKD1* mutations are known to be associated with a more severe disease course and progress to end-stage renal disease 20 years earlier than those with *PKD2* mutations and those with the latter mutation have fewer renal cysts [1]. It is hard to comment on this topic with only one patient with PKD2 mutation, however, our patient had a relatively low number of cysts. In two of our patients, no identifiable mutation was found, although they all had bilateral cysts and positive family history, and one additionally had VEO ADPKD. We may speculate that they had mutations that had not been identified yet or they may have mutations in *GANAB,* which cannot be checked in the genetic laboratory that we used to work with.

A consensus for the diagnosis, screening, treatment, or clinical care of children with ADPKD has been lacking until recently [2]. Diagnosing ADPKD is still challenging in children <15 years due to the lack of definitive criteria. Although not validated, a child with positive family history and a single cyst preferably >1 cm is generally assumed to have ADPKD [2]. Genetic analysis may also complete the diagnosis in a suspicious case, however may result in negative, as seen in our two cases. Recently, a new clinical practice guideline has been published on monitoring children with ADPKD [7]. 

Screening is generally not recommended in presymptomatic children due to insurability and emotional problems [8,9]. Diagnosing a child may cause difficulties in obtaining insurance in some countries. In addition, it has been demonstrated that 22% of the parents have fallen into depression and 62% feel guilty about their children’s disease [9]. In Kidney Disease Improving Global Outcomes (KDIGO) consensus guideline, screening presymptomatic children at risk with ultrasound or genetic testing is not recommended [10]. However, 3 different options for parents have been offered: not to screen; to screen as young as possible and disclose the results only to the parents, or to screen and disclose the results to the whole family [10]. In the European ADPKD Forum, genetic analysis has been defined as vital for diagnosis in children, but screening for at-risk children has not been discussed [2]. In the recent clinical guideline, discussing the potential harms and benefits of being diagnosed with the family and the child if possible, has been recommended [7]. In our series, patients were mostly brought to our clinic by their parents to learn whether the child has the disease, while in 19% of them, cysts were defined in their ultrasound coincidentally. However, family history was defined in almost all children when asked or cysts were demonstrated in the ultrasound (US) of their parents. 

No definite treatment has been established for children with ADPKD yet. That is another reason for ADPKD to be controversial to screen. Tolvaptan, statins, angiotension converting enzyme inhibitors (ACEIs), and mTOR inhibitors may be used in adults. Currently, V2-receptor antagonist tolvaptan is the only drug available for selected adults (<50 years, CKD stages 1-3a, rapidly progressing disease) to slow the progression of cyst development and renal insufficiency [11]. There is no enough evidence to use tolvaptan in children routinely, however, it has been used in a case with severe neonatal ADPKD [12]. The results of an ongoing phase IIIb double-blind, placebo-controlled study for tolvaptan treatment in children with ADPKD aged 4–17 years is pending [13]. Another treatment of choice in pediatric ADPKD is statins. In a 3-year randomized, double-blind, placebo-controlled study in children with ADPKD aged 8–22 years demonstrated that pravastatin may slow down the progression of kidney disease [14]. However, statin use in that age group is not safely advisable. A 5-year study on the effects of ACEIs in patients with ADPKD aged 4–21 years could not demonstrate any benefit on renal growth [15]. Due to the side effects and disappointing results in adults, mTOR inhibitors have no place for treatment in children with ADPKD as well [16]. Thus, none of our patients were on routine treatment. 

It is well-known that disease progression can be prevented by modifiable factors including proteinuria and hypertension [2,16]. In a metaanalysis, Marlais et al. have found that 20% of children with ADPKD have HT [17). They have observed that many children with ADPKD are not under regular follow-up and have recommended regular blood pressure control for all children at risk for ADPKD [17]. In KDIGO, it is recommended to screen children with a family history of ADPKD for HT from the age of 5 years and to repeat blood pressure measurements at 3 year intervals in case the patient is normotensive [10]. In the recent clinical guideline, blood pressure monitoring at least once every 2 years has been recommended for children aged 5 years and above and diagnosed with ADPKD or those at risk for developing the disease [7]. Target blood pressure limits have not been established well for children with ADPKD. In KDIGO, a goal blood pressure <90th percentile for age, sex, and height with renin-angiotensin-aldosteron system blockade as first-line treatment has been offered [10]. However, in a more recent study, a goal blood pressure ≤50th percentile with ACEIs has been reported to correlate with better eGFR and left ventricular mass index levels [14]. In addition to HT and proteinuria, glomerular hyperfiltration (GFH) is also a bad prognostic factor associated with a significantly faster renal function impairment and a higher renal growth rate [18]. Only 1 of our patients had HT and was put on ACEI, and 6 had orthostatic proteinuria. 26% of our patients had GHF and fortunately, none had a GFR lower than 90 mg/mL/1.73m2 during the follow-up period. Other nephro-urological problems in ADPKD include hematuria, pyuria, urinary tract infections, or nephrolithiasis [1]. Hematuria was detected in 12%, pyuria was detected in 10%, UTIs were detected in 14%, and urolithiasis was defined in 7% of the patients in our series. 

It is well-known that ADPKD is a systemic disorder causing cyst formation in other organs, mostly in the liver and pancreas, intracranial arterial aneurysms, cardiac valvular defects, inguinal and abdominal herniation, in addition to nephro-urological problems. Only one of our patients had hepatic cysts, and none had a splenic or pancreatic cyst. Screening children for intracranial aneurysms is uncertain. However, there is recent evidence that screening all patients regardless of the family history of aneurysms is cost-effective [19]. We have not routinely searched for intracranial aneurysms, however, 5 of our patients had MRA without any evidence of intracranial aneurysms. Echocardiography was performed in 12 of the patients that were normal in all but one with PFO. 

Height-adjusted total kidney volume (TKV) has been proven to be a strong and independent predictor for end-stage renal disease (ESRD) development in ADPDK [2]. We could not measure TKV in our patients. Instead, we have assessed the kidney sizes and cyst diameters obtained by ultrasonography and changes in them throughout the follow-up period. In adults, the total kidney and cyst volume enlargement rate slow down with age. In children, they are higher than adults and highest during puberty [1]. However, we could not exactly define such a correlation between the follow-up duration and cyst enlargement. In addition, age at diagnosis has no influence on cyst or kidney enlargement during the follow-up. 

The results of our study should be interpreted with its limitations. The first was the retrospective design of our study and the second was the limited number of cases. As recommendations for screening was unclear, not all our patients had evaluation for intracranial aneurysms, which is another limitation. Finally, not all the cases had genetic analysis and this prevented a definitive analyses about the correlations of mutations and the clinical entities. 

## 5. Conclusion

The majority of children with ADPKD in this study were found to have preserved renal functions and slight cyst enlargement during their follow-up supporting the debate about diagnosing children at-risk. However, early precautions have the potential to delay progression to ESRD and the patients may have different renal problems deserving closed follow-up. It would be more valuable to diagnose those children at-risk if ongoing trials define effective treatment strategies for cyst enlargement. 

## Funding information

The authors declare that no funding was received for the present study. 

## Ethical approval

The study was approved by the ethics committee of our institute (Date and IRB approval number: 27/03/2019;174). All procedures involving “Human beings” were conducted following the ethical standards and principles outlined in the Helsinki Declaration 2008. 

## Informed consent

Informed consent for participating in this study was not required.
